# WHO FCTC Article 19: Using the judicial system to fight tobacco

**DOI:** 10.18332/tid/130845

**Published:** 2020-12-01

**Authors:** Kelsey Romeo-Stuppy, Daniel Dorado, Davi Bressler, Deborah Sy, Amos Hausner

**Affiliations:** 1 Action on Smoking and Health, Washington, United States; 2 Corporate Accountability, Quito, Ecuador; 3 Procuradoria-Regional da União na 4ª Região, Brasilia, Brazil; 4 Global Center for Good Governance in Tobacco Control, School of Global Studies, Thammasat University, Rangsit, Thailand; 5 Independent researcher

**Keywords:** WHO FCTC Article 19, tobacco, tobacco control, tobacco laws

‘In the absence of the COVID-19 pandemic, many people in tobacco control worldwide would have been at the Hague, Netherlands, from 9–14 November for the 9th Conference of the Parties (COP9) of the WHO Framework Convention on Tobacco Control (FCTC), advocating for even stronger policies against the tobacco epidemic. The COP has been postponed to 2021, but the pandemic did not stop the global civil society from ‘virtually’ gathering to talk about the FCTC, where it is and where it is going.’

Engaging in litigation against the tobacco industry is intimidating. The industry has money, resources, confidence, and experience. As one example of the boldness of the tobacco industry, Philip Morris International sued the country of Uruguay to ‘make an example of Uruguay and intimidate other countries’^1^. As for money and resources, the United States Master Settlement Agreement between 46 U.S. states and the four largest tobacco companies is the largest settlement in the history of American litigation^2^; so while the stakes may be high, so is the potential reward.

However, Article 19 of the FCTC is intended to encourage the good guys, like ministries of health, to utilize the courts as well^3^. The Office of the Attorney-General of Brazil recently filed a lawsuit against the largest tobacco corporations in Brazil, seeking the recovery of healthcare costs related to the treatment of twenty-six tobacco related diseases. The claim also included compensation for future spending, and collective moral damages, as a consequence of the tobacco public health burden^4^. The suit can already be considered a success because it has increased public awareness of tobacco industry influence.

In addition to civil liability, Article 19 implores states parties to consider criminal liability. A case would be centered on one single product, the consumption of which is responsible for the demise of 1 in 2 users^5^ when consumed as intended and foreseen by the manufacturer. Further, there is no safe level of exposure to secondhand tobacco smoke^6^. These facts alone call for imposition of criminal liability, from homicide to reckless endangerment. Article 19 provides the obligatory platform for such prosecution and claims, and it can well be the mechanism for the result: the days of the tobacco industry as we know it are numbered.

This type of action is beginning to be utilized in tobacco control, led by the example in the Netherlands. The Dutch group, Sick of Smoking, encouraged a prosecutor to bring criminal charges against the tobacco corporations for fraud, manslaughter, and attempted murder. Unfortunately, the appeals court declined to force the prosecutor to file charges. However, this case was a huge victory in many ways. It garnered an amazing amount of press and attention, and had over 30000 people sign on in support^7^. It changed public opinion around the tobacco industry and was a clear example to other tobacco control advocates that criminal liability is a feasible option.

Tobacco litigation has inspired the use of litigation in other causes as well. For example, in the case of climate change, many advocates are now encouraging pursuing legal remedies to require corporate polluters to pay^8^. There is a working group in the U.N. system that is currently drafting a legally binding instrument to regulate the activities of transnational corporations and other business enterprises^9^. The practices and policies of Article 19, among other examples, were considered by some of the working group members^10^. It is fantastic to see that Article 19 of the FCTC is a model for addressing corporate malfeasance in many different areas.

Further, the ‘traditional’ concept of liability is being expanded into a new arsenal of options, including utilizing administrative liability, expanding the scope into quasi-judicial and non-judicial systems, pursuing justice for victims, and identifying compensation mechanisms that work. Article 19 gives us the framework, but it is up to tobacco control advocates to use that framework to create lasting change.
